# The enzyme activity of histone deacetylase 8 is modulated by a redox-switch

**DOI:** 10.1016/j.redox.2018.09.013

**Published:** 2018-09-27

**Authors:** Niklas Jänsch, Christian Meyners, Marius Muth, Aleksandra Kopranovic, Olaf Witt, Ina Oehme, Franz-Josef Meyer-Almes

**Affiliations:** a Department of Chemical Engineering and Biotechnology, University of Applied Sciences Darmstadt, Haardtring 100, 64295 Darmstadt, Germany; b Preclinical Program, Hopp Children's Cancer Center at NCT Heidelberg (KiTZ), Germany; c Clinical Cooperation Unit Pediatric Oncology, German Cancer Research Center (DKFZ), INF 280, D-69120 Heidelberg, Germany; d Department of Pediatric Oncology, Hematology and Immunology, University Hospital Heidelberg, Heidelberg, Germany; e German Cancer Research Consortium (DKTK), Germany

**Keywords:** HDAC8 stability, Redox kinetics, Redox signaling, NOX, Disulfide bond, ROS, Hydrogen peroxide

## Abstract

Enzymes from the histone deacetylase (HDAC) family are highly regulated by different mechanisms. However, only very limited knowledge exists about the regulation of HDAC8, an established target in multiple types of cancer. A previous dedicated study of HDAC class I enzymes identified no redox-sensitive cysteinyl thiol in HDAC8. This is in contrast to the observation that HDAC8 preparations show different enzyme activities depending on the addition of reducing agents. In the light of the importance of HDAC8 in tumorigenesis a possible regulation by redox signaling was investigated using biochemical and biophysical methods combined with site directed mutagenesis. The occurrence of a characteristic disulfide bond under oxidizing conditions is associated with a complete but reversible loss of enzyme activity. Cysteines 102 and 153 are the integral components of the redox-switch. A possible regulation of HDAC8 by redox signal transduction is suggested by the observed relationship between inhibition of reactive oxygen species generating NOX and concomitant increased HDAC8 activity in neuroblastoma tumor cells. The slow kinetics for direct oxidation of HDAC8 by hydrogen peroxide suggests that transmitters of oxidative equivalents are required to transfer the H_2_O_2_ signal to HDAC8.

## Highlights

• The enzyme activity of HDAC8 is regulated by a redox-switch.
• The structure of the inactive oxidized state is thermodynamically stabilized.
• C102 and C153 are the integral components of the redox-switch in HDAC8.
• NOX inactivation lowers intracellular H_2_O_2_ and increases HDAC8 activity.
• Slow oxidation kinetics suggests indirect transduction of the H_2_O_2_ signal in cells.

## Graphical abstract

**Figure:**
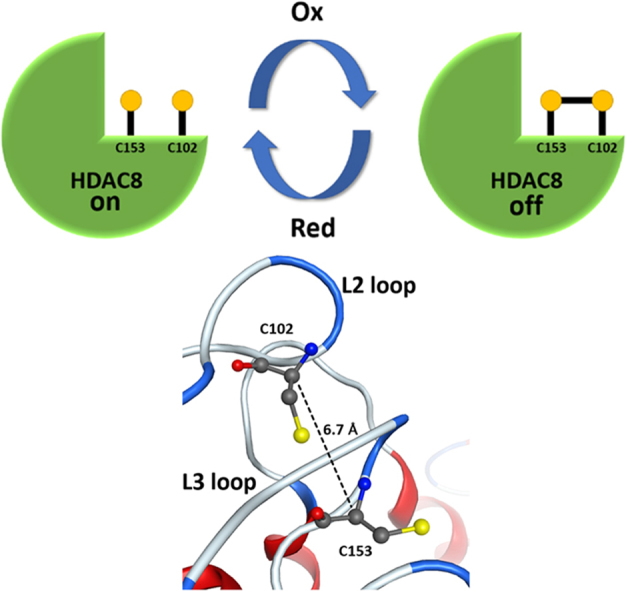

## 1 Introduction

Histone deacetylases (HDACs) have emerged as promising targets for chemotherapeutic intervention in cancer, neurodegenerative and immune disorders [1], [2], [3]. The HDAC enzyme family is subdivided into class I (HDAC1–3 and 8), class IIa (HDAC4,5,7 and 9), class IIb (HDAC6 and 10), class III (sirtuins SIRT1–7) and class IV (HDAC11). The zinc dependent HDACs of class I, IIa/b and IV operate with a different mechanism than the sirtuins in class III which require NAD^+^ for catalysis. HDAC8 is an established target for T-cell lymphoma and neuroblastoma and overexpressed in other tumors [4]. HDAC8 is found in the nucleus as well as the cytoplasm which is in line with an increasing number of identified substrates (SMC3, ERR-α(SMC3, E, RAI1, MLL2, p53, Cortactin) [5], [6], [7] and interaction partners (CREB/PP1, hEST1B, Inv(16) fusion protein, DEC-1, Hsp20, α-actin) [8], [9], [10], [11], [12] in both compartments. Consequently, the physiological role of HDAC8 in cells is complex and needs further elucidation but it is clearly linked to relevant cancer mechanisms. HDAC8 activity is negatively regulated upon phosphorylation by cyclic AMP dependent protein kinase at position serine 39 [13]. Moreover, Fierke et al. suggested that HDAC8 could be also regulated by metal switching *in vivo* [14]. Redox control of epigenetic processes has been proposed as a general principle to allow the cell to adapt to a changing environment. During the last years, it became clear that redox-sensitive proteins like peroxiredoxins serve not only as cellular self-defense against oxidative stress but can also act as redox relay for specific H_2_O_2_ signaling in cells [15]. Several HDACs were shown to be redox-regulated. The class IIa HDAC4 has a redox-switch that controls shuttling between the nucleus and the cytoplasm [16]. Moreover, HDAC6 has been shown to interact with peroxiredoxins [17]. In another study, class I HDACs -1, -2 and -3 but not HDAC8 were proposed to be redox-sensitive and a putative redox-switch was identified using chemical probes [18]. However, we and others observed that HDAC8 activity depends on the enzyme preparation conditions [19]. Inspired by the additional finding that inactive oxidized HDAC8 can be retransformed into active enzyme by the addition of Tris(2-carboxyethyl)phosphine (TCEP) or β-mercaptoethanol (β–ME), we initiated a comprehensive study to elucidate the mechanism of the redox-switching behavior of HDAC8. We provide unambiguous evidence that HDAC8 has a reversible thiol/disulfide redox-switch involving C153 and C102 and that the oxidized state of HDAC8 is thermodynamically more stable than the corresponding reduced state. The potential physiological relevance of redox-regulation of HDAC8 is demonstrated by the inhibition of endogenously produced H_2_O_2_ in neuroblastoma cells.

## 2 Materials and methods

### 2.1 Materials

All reagents and solvents were purchased from Sigma, Bachem, Roth, and used with further purification only if necessary. 9,9,9-trifluoro-8-oxo-N-phenyl-nonanamide (SATFMK) was prepared as described [20]. To minimize artificial oxidation of free thiols all measurements were performed in degassed buffer solutions. To prevent oxidation of enzyme during storage, enzymes were stored in the presence of 1 mM TCEP. TCEP was removed immediately before measurements by gel permeation chromatography with Zeba Spin Desalting Columns 7K MWCO (Thermo Scientific).

### 2.2 HDAC8 mutant variants

Mutant HDAC8 variants were generated using splicing by overlap extension PCR (SOE-PCR) with the following Primers:

HD8_BamHI_rev: 5′-AGGTGGATCCTTAAACAACGTGCTTCAGATTGCC-3′,
HD8_NdeI_for: 5′-GCGCATATGGAGGAGCCGGAGGAG-3′,
HD8_C102S_for: 5′-GGGCTAGGTTATGACTCCCCAGCCACTGAAGGGATA-3′,
HD8_C102S_rev: 5′-TATCCCTTCAGTGGCTGGGGAGTCATAACCTAGCCC-3′,
HD8_C153S_for: 5′-GATGAAGCATCTGGTTTTTCTTATCTCAATGATGCT-3′,
HD8_C153S_rev: 5′-AGCATCATTGAGATAAGAAAAACCAGATGCTTCATC-3′.
DNA sequencing was performed at the sequencing service at the LMU Munich with the cycle, clean and run (BigDye v3.1) protocol.

### 2.3 HDAC8 expression and purification

pET14b vector (Novagen, EMD Millipore) containing codon-optimized human HDAC8, fused to a His_6_ SUMO tag, was used to express HDAC8 in E. coli (BL21) DE3 pLysS. Cells were harvested by centrifugation for 10 min at 8000*g* and 4 °C and resuspended in lysis buffer (pH 8.0) containing 150 mM KCl, 50 mM Tris, 5 mM imidazole, 5 mM DTT, 1x HALT protease inhibitor cocktail (Thermo Scientific) and 5 µg/mL DN*Ase*I. The cell suspension was sonicated and lysates were clarified by centrifugation at 18,000*g* for 30 min at 4 °C and sterile filtration. The filtrate was subsequently added to a 5 mL column volume of cOmplete His tag purification resin (Roche), equilibrated with immobilized metal affinity chromatography (IMAC) buffer A (pH 8.0) containing 150 mM KCl, 50 mM Tris and 5 mM imidazole. After washing with 50 mL of the same buffer His_6_-SUMO-HDAC8 was eluted with IMAC buffer B (pH 8.0) containing 150 mM KCl, 50 mM Tris and 100 mM imidazole. Subsequently 10 µg/mL His_6_ tagged SUMO-Protease was added to the eluted HDAC8 fusion protein. Cleavage of His6-SUMO tag occurred overnight whilst dialyzing against 25 mM Tris, 50 mM NaCl and 5 mM ß-ME (pH 8.0) at 4 °C. Then His6-SUMO tag and SUMO-Protease were removed by a second IMAC with AIC buffer A (pH 8.0) containing 25 mM Tris and 50 mM NaCl and 5 mM imidazole. HDAC8 containing flow through was concentrated and further purified on a strong anion exchanger (Bio-Scale Mini Macro-Prep High Q 5 mL Cartridge, Biorad). After a washing step using AIC buffer A HDAC8 was eluted using AIC buffer B (pH 8.0) containing 25 mM Tris and 1 M NaCl. 5 mM DTT was added to prevent oxidation and remove possible ß-ME cysteine adducts. The final purification step included size exclusion chromatography with a HiLoad Superdex 75 material (GE) equilibrated with GPC Puffer (pH 8.0) containing 150 mM KCl and 50 mM Tris. The protein containing fractions were collected and concentrated. Glycerol and TCEP were added to final concentrations of 25% and 1 mM and protein was stored at −20 °C. We typically obtained 3–5 mg HDAC8 from 1 L culture.

### 2.4 Enzyme activity assays

The activity of all HDAC8 variants was determined in black half area 96-well microplates (Greiner bio-one, Germany) by a colorimetric assay described by Wegener et al. [21]. HDAC8 (10 nM) was incubated with indicated concentrations of H_2_O_2_ for 1 h at 30 °C in HDAC8 assay buffer containing 25 mM Tris-HCl, 75 mM KCl and 0.001% Pluoronic F-127 pH 8.0. Excess H_2_O_2_ was quenched by the addition of 5.6 µg/mL freshly dissolved catalase. The reaction was initiated by the addition of 10 µM of the substrate Boc-Lys(tri-fluoroacetyl)-7-amino-4-methylcoumarin (Boc-Lys(TFA)-AMC). After incubation for 60 min, the reaction was stopped by the addition of 1.67 µM SATFMK and the deacetylated substrate was converted into a fluorescent dye (AMC) by the addition of 0.42 mg/mL trypsin. Measurements were performed in a fluorescence microplate reader (PHERAstar FS, BMG LABTECH). The data was analyzed with GraphPad Prism version 6.01.

### 2.5 Electrophoretic mobility shift assay (EMSA)

For the analysis of disulfide bond formation via migration change on non-reducing SDS-PAGE 5 µM of the respective HDAC8 variant was treated with increasing concentrations of H_2_O_2_ (0–10 mM) in redox buffer containing 20 mM NaH_2_PO_4_, 20 mM Na_2_HPO_4_, 150 mM NaCl and 5 mM EDTA pH 7.0. After 1 h incubation at room temperature excess H_2_O_2_ was quenched by the addition of 10 µg/mL catalase and free thiole groups were blocked by the addition of 8.3 mM NEM to prevent unwanted rearrangements of disulfide bonds followed by an incubation period of 30 min at room temperature. Finally, 4x non-reducing sample buffer was added containing 8% SDS, 250 mM Tris-HCL (pH 6.8), 40% Glycerol and 0.02% Bromophenol blue. The samples were denaturated for 5 min at 95 °C and cooled on ice. Subsequently, SDS-PAGE was performed on 12.5% gels at 200 V. Gels were stained with Coomassie brilliant blue solution.

### 2.6 Determination of the redox-potential between Cys_102_ and Cys_153_

A codon optimized gene was purchased, with every cysteine (C28, C125, C131, C244, C275, C287, C314 and C352) changed to serine except Cys_102_ and Cys_153_. This HDAC8_lowC_ variant was expressed and purified as described above. At first a 2-fold serial dilution of 20 mM GSH was performed by keeping GSSG constant at 2 mM in a volume of 20 µL in buffers with three different pH-values (HEPES 100 mM, EDTA 100 µM, pH 7.0; Tris 100 mM, EDTA 100 µM, pH 8.0; CHES 100 mM, EDTA 100 µM, pH 9.0). Immediately after preparing the solutions 20 µL of the mutant HDAC8 was added to each mixture and kept overnight under nitrogen atmosphere to prevent oxygen oxidation. After reaching the equilibrium 5 µL TCA (100% (w/v)) was added to each sample and protein was precipitated for 20 min at −20 °C followed by 10 min centrifugation at 18,000*g* at 4 °C. The supernatant was removed, and the pellet resuspended by shaking in 30 µL redox-buffer containing 1 mM NEM for 30 min at 30 °C. After alkylation of nascent thiols 10 µL 4x non-reducing sample buffer was added to each sample followed by denaturation for 5 min at 95 °C. Samples were subjected to non-reducing SDS-PAGE and blotted on a PVDF Membrane (Merck, Millipore). Protein was detected using primary antibody for HDAC8 (E5, Santa Cruz biotechnology) and dye-labelled IRDye 800CW goat anti-mouse antibody (LiCor). Band intensities were quantified using the Image Studio Lite Software (LiCor). The redox-potential was determined at pH 7.0, 8.0 and 9.0. The dependency of the standard redox-potential from pH is described by the following general equation involving two protons:

### 2.7 Differential scanning fluorimetry to determine protein stability

Protein melting points were determined using a Prometheus NT.48 instrument from Nanotemper. 12 µM of the respective HDAC8 variant was treated with increasing concentrations of H_2_O_2_ (ranging from 0 to 5 mM) in redox buffer containing 20 mM NaH_2_PO_4_, 20 mM Na_2_HPO_4_, 150 mM NaCl and 5 mM EDTA pH 7.0. After 1 h incubation at room temperature, 10 µg/mL catalase was added to eliminate excess H_2_O_2_ and stop the oxidation process. The measurement was performed by increasing the temperature to 95 °C with a heating rate of 1 °C per minute and simultaneous detection of the fluorescence ratio of 350 nm/330 nm. The melting points were determined from the minimum of the first derivative of the function.

### 2.8 CD-spectroscopy

Spectra were collected by using a Jasco j-815cd spectropolarimeter (Easton, MD) equipped with a temperature controlling device in a quartz cell with a path length of 0.1 cm. Before experiments, HDAC8 was desalted by GPC with Zeba Spin Desalting Columns 7K MWCO (Thermo Scientific) equilibrated with CD buffer (pH 8.0) containing 5 mM Tris, 0.5% glycerol, 15 mM KCl and 0.1 mM DTT. Oxidized HDAC8 was generated by the addition of 5 mM H_2_O_2_ for 1 h at room temperature and desalted against CD buffer without DTT. The final protein concentration was 5 µM. Spectra were recorded in the far-UV from 260 to 195 nm and were the average of five scans.

### 2.9 Kinetics of HDAC8_wt_ oxidation

To evaluate the kinetics of disulfide bond formation between the amino acids C102 and C153, 2.5 µM HDAC8_wt_ was treated with various concentrations of H_2_O_2_ (ranging from 0 to 2.5 mM) and 200 µM Fluor-de-Lys HDAC substrate in potassium phosphate buffer (pH 7.6) containing 30 mM potassium phosphate, 100 mM KCl and 5% glycerol at 20 °C. Aliquots were removed at the indicated time periods and the reaction was quenched by the addition of 100 µM N‐hydroxy‐N′‐phenyloctanediamide (SAHA) and 0.04 mg/mL catalase after oxidation, 0.8 mg/mL Trypsin was added to generate fluorescent AMC for 15 min at 30 °C. The data was fitted using an exponential function in Graph Pad Prism and the observed pseudo first order rate constants were plotted against the different H_2_O_2_ concentrations. The second order rate constant of oxidation was determined from the slope of the plot.

### 2.10 Reversible redox modulation of HDAC8_wt_ activity

H_2_O_2_ and TCEP were added successively to a solution of fully reduced HDAC8_wt_ (2.5 mg/ml in 25 mM Tris-HCL, 75 mM KCl and 0.001% Pluronic) to achieve alternating reducing and oxidizing conditions. HDAC8_wt_ was incubated with H_2_O_2_ for 45 min at 25 °C and TCEP for 60 min at 4 °C, respectively, while shaking at 650 rpm in a Thermomixer from Eppendorf. The oxidation process was stopped by adding 0.1 mg/mL catalase from bovine liver (Sigma-Aldrich). As control untreated HDAC8_wt_ was supplemented by corresponding volumes of buffer. Aliquots were taken from each mixture to measure HDAC8_wt_ activity. The samples for the enzyme activity assay (see above) of HDAC8_wt_ were pretreated as follows: TCEP was removed and the buffer renewed using Zeba™ Spin Desalting Columns, 7K MWCO (Thermo Scientific). The redox modulation of HDAC8_C102S/C153S_ activity was determined under the same conditions.

### 2.11 Western blot analysis

Western blot analysis was performed as described previously. [22] The following antibodies were used for detection: anti-acetyl-SMC3 (provided by Prof. K Shirahige, University of Tokyo, Tokyo, Japan) and anti-β-actin (clone AC-15; Sigma).

### 2.12 Determination of the Michaelis-Menten-parameters

10 nM (HDAC8_wt_, HDAC8_C102S_) and 100 nM (HDAC8_C153S_, HDAC8_C102S/C153S_) were incubated with increasing concentrations of Boc-Lys(TFA)-AMC for 5 min at 21 °C in HDAC8 assay buffer containing 25 mM Tris-HCl, 75 mM KCl and 0.001% Pluronic F-127 pH 8.0. After incubation 0.5 mg/mL Trypsin and 50 µM SAHA was added to stop enzyme reaction and generate fluorescent AMC signal. Additionally, a series of varying concentrations AMC in the buffer described above was measured and RFU was plotted against AMC concentration. Resulting fluorescent signal per µM AMC was observed by the slope of linear regression.

### 2.13 NOX inhibition

BE(2)-C cells were incubated for 6 h with 5 and 10 µM NADPH oxidase (NOX) inhibitor VAS2870 [23]. Whole cell lysates were blotted against SMC3 and normalized by the actin concentration of the same sample.

## 3 Results and discussion

### 3.1 Enzyme activity of HDAC8 correlates with its redox state

There is a large number of publications about the modulation of HDAC8 enzyme activity by inhibitors or incorporated metal ions [14], [24]. In particular, the identification of N-acetylthiourea compounds as putative activators of HDAC8 raised considerable attention [25]. However, Toro et al. demonstrated that the activation effect was essentially due to enzyme preparations with inherently low activity [19]. Toro et al. suspected that the preparation conditions in the study of Singh et al. might have led to partially metal-depleted HDAC8 or stabilized a particular inactive conformation of the enzyme, which could be possibly activated by the N-acetylthiourea compounds. However, the authors did not identify the specific mechanism of activation, nor did they take into consideration possible redox effects as a causal factor for the postulated preparation effects.

**Fig. 1:**
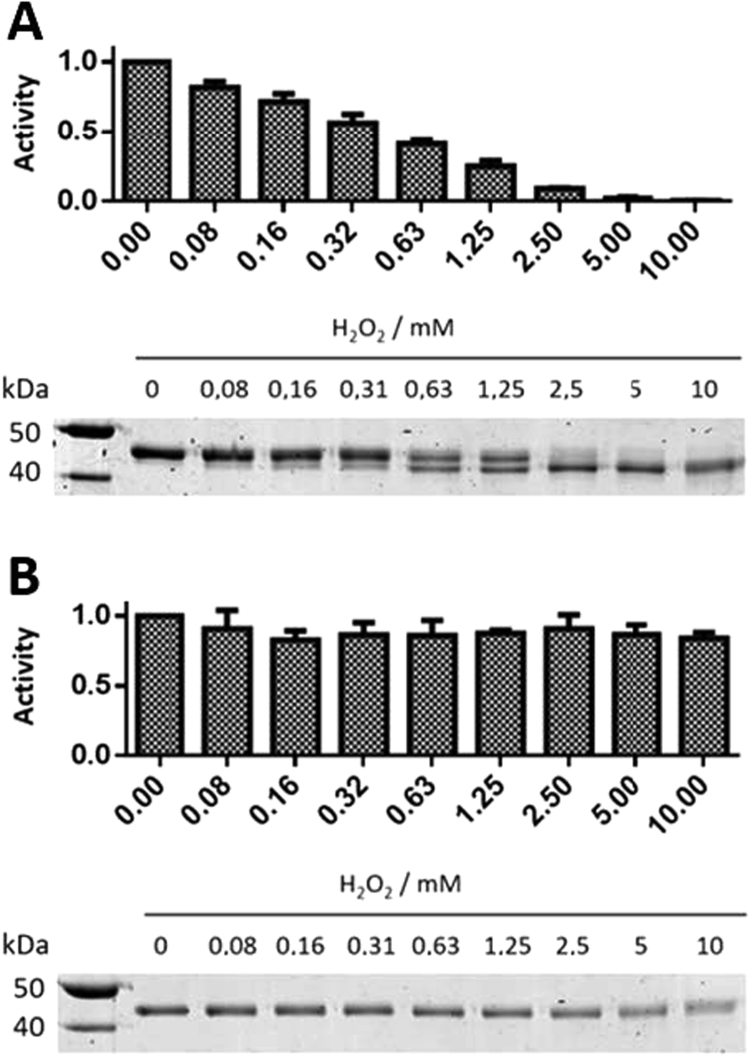
Enzyme activity (upper panel) and EMSA (lower panel) of (A) HDAC8_wt_ and (B) double-mutant HDAC8_C102S/C153S_ in the presence of increasing amounts of H_2_O_2_. The enzyme activity test exploited the conversion of Boc-Lys(TFA)-AMC in the first step followed by the addition of trypsin to release fluorescent AMC. Gels were stained with Coomassie brilliant blue. The most left gel slot contains a ruler with indicated molecular weights. The upper band represents the reduced and the lower band the oxidized form of HDAC8_wt_. The mean and standard deviations were calculated from four independent experiments each performed in triplicates.

We and others observed that the enzyme activity of HDAC8 decreases rapidly even if stored refrigerated at 4 °C. We also found that part of the activity of HDAC8_wt_ can be recovered by the addition of reducing agents like DTT or TCEP. To elucidate a putative redox-sensitivity of HDAC8, we initiated a comprehensive study combining a variety of complementary biochemical, biophysical and cell-based approaches. Analysis of HDAC8_wt_ under non-reducing conditions revealed that enzyme activity is lost depending on hydrogen peroxide (H_2_O_2_) concentration (Fig. 1A). The oxidized form of HDAC8_wt_ with disulfide bonds adopts a more constrained structure after denaturation and thus runs significantly faster in a non-reducing SDS-PAGE than the reduced form of HDAC8_wt_ (lower panel of Fig. 1A). Increasing amounts of H_2_O_2_ shift the redox-equilibrium progressively to the oxidized state of HDAC8_wt_. The percentage of the reduced state of HDAC8_wt_ correlates perfectly with enzyme activity (Fig. 1A).

In contrast, the double mutant HDAC8_C102S/C153S_ is essentially insensitive to H_2_O_2_ (Fig. 1B). The enzyme activity remains unchanged in the presence of up to 10 mM H_2_O_2_ and no additional band indicating a disulfide bridge becomes visible in the corresponding non-reducing gel.

### 3.2 The redox state-dependent enzyme activity of HDAC8 is reversible

**Fig. 2:**
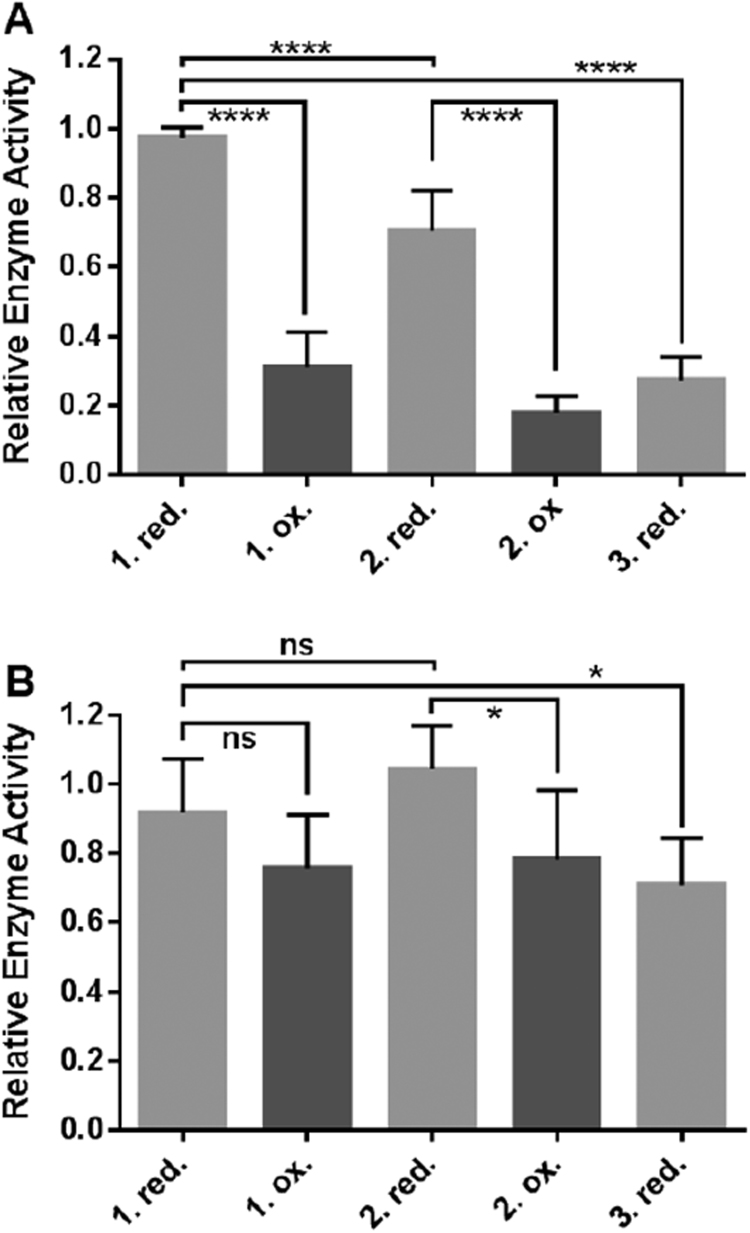
Reversibility of redox modulation of the enzyme activity of (A) HDAC8_wt_ and (B) HDAC8_C102S/C153S_. The enzyme activity of the same HDAC8 sample after subsequent alternating addition of slight stoichiometric excesses of TCEP (red.) and H_2_O_2_ (ox.) is shown, respectively. The data represent means and standard deviations (HDAC8_wt_: n = 15, HDAC8_C102S/C153S_: n = 6). Statistical significance was determined by an unpaired *t*-test. ns: not significant (p > 0.05), *: 0.001 < p < 0.05 and ****: p < 0.0001.

The enzyme activity of oxidized HDAC8_wt_ can be reversed repeatedly by adding reducing agent (Fig. 2A). However, overdosing and long exposure to H_2_O_2_ produces overoxidation of HDAC8_wt_ that cannot be reversed. During this process, thiol groups are in general irreversibly oxidized further to sulfinic and sulfonic acid [26], [27]. Overoxidation was prevented to a great extent by the addition of catalase immediately after H_2_O_2_ mediated oxidation. Nevertheless, the maximum enzyme activity decreased significantly (p < 0.0001) after each redox-cycle presumably due to unavoidable residual overoxidation (Fig. 2A). To determine whether the progressive loss of enzyme activity was due to oxidation elsewhere in the protein or specific for the C102/C153 redox switch, we repeated the experiment with HDAC8_C102S/C153S_ double mutant protein (Fig. 2B). The progressive loss of enzyme activity during the redox cycles was clearly less pronounced for the HDAC8 _C102S/C153S_ variant lacking the redox switch than for HDAC8_wt_ (71% remaining activity for HDAC8 _C102S/C153S_ and 27% remaining activity for HDAC8_wt_). Therefore, the postulated overoxidation process appears to be predominantly specific for the redox switch. However, other less dominant irreversible deactivation pathways cannot be completely ruled out.

**Fig. 3:**
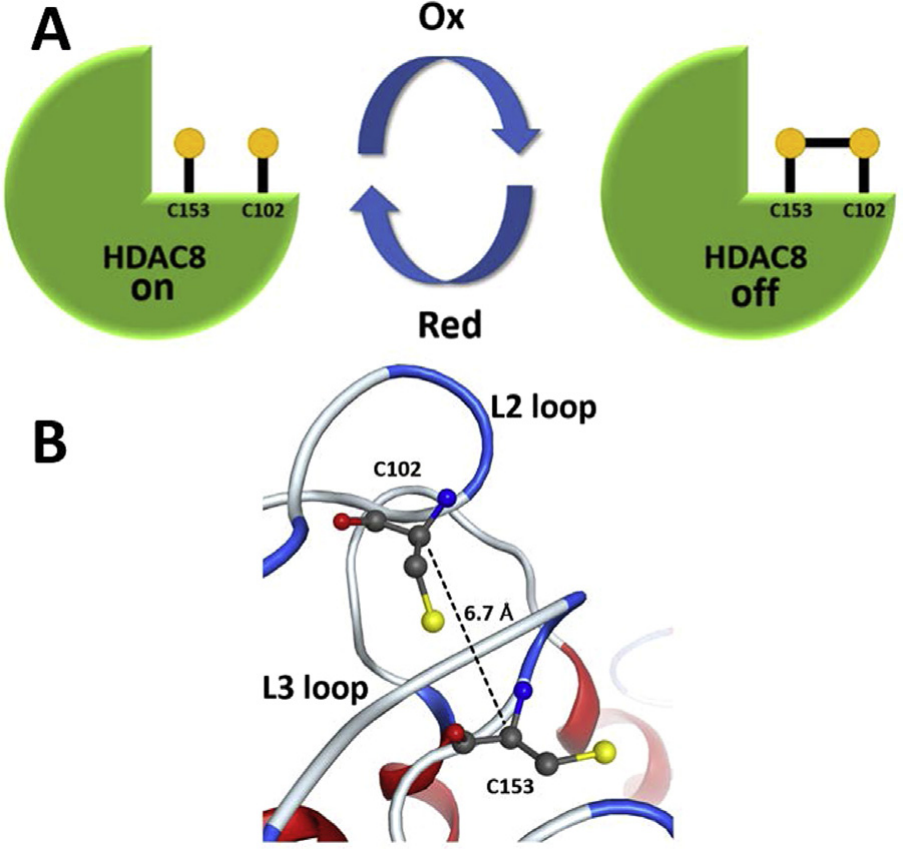
A) Scheme of redox-switch: HDAC8_wt_ is inactivated by H_2_O_2_ induced reversible formation of a disulfide bridge between C153 and C102. B) Arrangement of C102 and C153 in two adjacent flexible loops, L2-loop containing C102 and L3-loop containing C153, showing a distance that enables the formation of a disulfide bond (PDB-ID: 1T64).

The redox switch of HDAC8 consists of C102 and C153. In a previous study, no redox-sensitive cysteines were identified in HDAC8 using cyclopentenone prostaglandins as tool compounds to identify redox-sensitive cysteine residues by alkylation [18]. In contrast, HDAC1,-2 and -3 were shown to contain redox-responsive cysteine residues in the same study. The alkylated redox-sensitive cysteines in HDAC1 were found at two different positions, where HDAC8 showed no homologous cysteine. After that study, several X-ray structures of class I HDACs 1, -2 and -3 were published. A closer look for example at the crystal structures of HDAC1 (PDB-ID: 4BKX) reveals that C273 sits on the surface of HDAC1 and is more than 16 Å away from the closest other cysteine. However, this cysteine might be involved in the formation of dimers upon oxidation. The second cysteine, C261, is buried in the interior of the properly folded protein and appears virtually inaccessible for larger alkylating agents such as cyclopentenone prostaglandins. It appears also reasonable that these bulky alkylating agents could have failed to modify the redox-sensitive C102 and C153 in HDAC8 due to sterical hindrance. The analysis of the X-ray structure of HDAC8 (PDB-ID: 1T64) reveals that C153 and C102 are in close proximity to each other (distance between Cα-atoms: 6.7 Å) (Fig. 3) and in contact with the active site.

According to the crystal structure (PDB-ID: 1T64) at least one of these cysteines, located in the L3-loop lining the major active site binding pocket (C153) or the neighboring L2-loop (C102), should be accessible by H_2_O_2_ and eventually form a disulfide bond with the other cysteine. Single exchange of C102 against serine has no significant influence on the catalytic efficiency of about 13,000 M^−1^ s^−1^ in HDAC8_wt_ (Table 1, Fig. S1). In contrast, the HDAC8_C153S_ and HDAC8_C102S/C153S_ mutants show a 4–9 fold lower catalytic efficiency than the wildtype enzyme indicating that C153 but not C102 plays a certain role in the catalytic mechanism of HDAC8 (Table 1, Fig. S1). However, substitution of C153 by serine does not abolish catalytic activity completely.

**Table 1:** Michaelis-Menten-parameters of HDAC8 variants.

Enzyme	K_M_ (µM)	k_cat_ (s^−1^)	k_cat_ K_M_^−1^ (M^−1^ s^−1^)
HDAC8_wt_	96 ± 23	1.22 ± 0.14	13,000 ± 4400
HDAC8_C153S_	179 ± 42	2.08 ± 0.24	3200 ± 1200
HDAC8_C102S_	116 ± 27	0.58 ± 0.08	18,000 ± 6200
HDAC8_C102S/C153S_	193 ± 32	0.38 ± 0.04	2000 ± 520

Means and standard deviations are provided, n = 3.

Furthermore, if the cysteines (C102, C153) involved in the putative redox-switch are exchanged with serine, the residual enzyme activity of the double mutant HDAC8_C102S/C153S_ becomes insensitive to treatment with H_2_O_2_ (Fig. 1B). Consistently, no second band that is indicative for the oxidative state of HDAC8 occurs in the non-reducing SDS-PAGE gel upon treatment with hydrogen peroxide. Taken together, these results clearly prove that C102 and C153 constitute a redox-switch in HDAC8.

### 3.3 The reduced form of HDAC8_wt_ is thermodynamically stabilized upon oxidation

The overall structure of oxidized HDAC8 shows no major deviations from its reduced form. The CD-spectra of the non-functional oxidized and the functional reduced form of HDAC8_wt_ show no significant differences (Fig. S2). Similarly, the spectra of mutant proteins HDAC8_C102S_, HDAC8_C153S_ and HDAC8_C102S/C153S_ resemble that of wildtype HDAC8. Thus, oxidation of HDAC8 is not associated with major rearrangements of its overall structure. To investigate the effect of oxidation on protein stability, Differential Scanning Fluorimetry analyses of HDAC8_wt_ were performed. Increasing H_2_O_2_ concentrations cause a continuous shift of melting temperature from 42.27 °C of the reduced form to 44.14 °C (Table 2, Fig. S3A/B) indicating that the oxidized form of the protein is considerably more stable than the reduced form. This is in agreement with the formation of a stabilizing disulfide bond between C102 and C153. Consistently, the functional reduced HDAC8_wt_ and mutant variants of HDAC8 with at least one of the cysteines C102 or C153 exchanged against serine show similar melting temperatures significantly distinct from that of inactive oxidized HDAC8_wt_ (Table 2, Fig. S3C/D). The L2-loop is highly unordered in crystal structures of unbound HDAC8 and becomes more ordered upon binding to small molecule ligands [28]. Moreover, ligand binding and the enzyme activity of HDAC8 largely depend on the conformation and flexibility of the L1- and L2-loops [29], [30], [31], [32]. In fact, major movements of these loops and the side chain of F152 in the L3-loop are responsible for a transition from a wide-open state (PDB-ID: 1VKG) of the active site pocket into a sub-open state with a second transient binding pocket (PDB-ID: 1T64) and then into a closed state (PDB-ID: 1T69) [33]. These three representative crystal structures of HDAC8 were analyzed using the TRAnsient Pockets in Proteins (TRAPP) software platform developed in the laboratory of Rebecca Wade. The results confirmed that the L2- and L3-loop of HDAC8 are regions of increased flexibility (Fig. S4), which is in agreement with the experimentally observed large structural variations around the active site binding pocket of HDAC8. In a structurally highly related HDAC homolog HDAH from *Bordetella/Alcaligenes,* the L2- and L3-loop are interconnected and stabilized by a network of hydrogen bonds involving the pivotal amino acid T101. Substitution of T101 by alanine interrupts the strong interaction between both loops and leads to a dramatically increased flexibility of the L2-loop changing the mode of ligand interaction [34]. The formation of a disulfide bridge between C153 (L3-loop) and C102 (L2-loop) in HDAC8 introduces a covalent link between the L2- and L3-loop, which is also supposed to reduce the intrinsic flexibility of these loops adjacent to the catalytic site pocket. Wang et al. as well as Whitehead et al. postulated a mechanism of acetate release involving major conformational changes in the acetate release channel with R37 and W141 (numbering refers to HDAC8) acting as gate keepers [35], [36]. The crucial importance of R37 for catalysis and function of the internal acetate release cavity of HDAC8 was experimentally confirmed by Haider et al. [37]. In summary, it can be concluded that the enzyme activity of HDACs and in particular HDAC8 is closely related with conformational flexibility at the active site binding pocket as well as the internal acetate release channel. The decreased flexibility at the active site upon oxidation of the redox-switch and covalent linkage of the L2- and L3-loop is suggested to contribute to the observed concomitant total loss in enzyme activity.

**Table 2:** Melting temperatures of HDAC8_wt_ and mutant enzymes in the absence or in the presence of indicated concentrations of H_2_O_2_. Melting temperatures (T_m_)are shown as means with standard deviations, n = 3.

HDAC8_wt_		
H_2_O_2_ (mM)		T_m_ (°C)
5.00		44.14 ± 0.33
2.50		43.96 ± 0.02
1.25		43.60 ± 0.08
0.63		43.26 ± 0.06
0.31		42.92 ± 0.03
0.16		42.57 ± 0.07
0.08		42.42 ± 0.05
0		42.27 ± 0.08
HDAC8_C102_		
0	42.91 ± 0.05	
HDAC8_C153_		
0	42.38 ± 0.01	
HDAC8_C102S/C153S_		
0		42.26 ± 0.03

### 3.4 The amino acids C102 and C153 are conserved in class I HDACs

C153 in HDAC8 is conserved throughout all members of human HDACs from classes I, IIa and IIb, whereas C102 is only conserved in class I HDACs (Fig. S5). In class IIa HDACs 4, 5, and 9 this cysteine is replaced by serine with the exception of HDAC7, where cysteine is replaced by alanine. Although the multiple sequence alignment suggests also a cysteine in the second domain of HDAC6 at the position of C102 (HDAC8), this is clearly an artifact of the heuristic alignment algorithm, because an alignment of crystal structures of HDAC1, HDAC8 and HDAC6 proves that I569 and no cysteine of HDAC6 matches C102 of HDAC8 (Fig. S6). Therefore, other HDAC class I members are also potentially regulated through a redox-switch corresponding to C102/C153 in HDAC8.

### 3.5 Standard redox-potential of C102/C153 switch

Since the ratio of the oxidized and reduced form of HDAC8 can be easily obtained from a non-reducing gel, the redox-potential of the molecular switch of HDAC8 could be determined from redox titrations (Fig. S7). The redox-potential of the C102/C153 redox-switch shows a linear dependency on pH and decreases from −218 mV at pH 7.0 to −340 mV at pH 9.0. ese redox-potential differences per pH-unit are in excellent agreement with 60.2 mV calculated from the corresponding equation under Materials and Methods at 30 °C involving two protons. The very good correlation between the experimental and calculated pH-dependency of the redox-potentials in HDAC8_lowC_ confirms that the C102/153 couple is exclusively responsible for redox-switching of HDAC8. Typical redox-potentials of the GSH/GSSG couple observed in cells range from −170 mV [38], [39] to −258 mV [40].

HDAC8 can exist in both redox forms under these conditions. Interestingly, the standard redox potential of rapidly proliferating cells appears to be more negative than for differentiated, slowly proliferating cells [40]. Therefore, the ratio of the reduced active and the oxidized inactive form of HDAC8 in faster proliferating cancer cells (E = −258 mV) would be about 20:1 according to the Nernst equation. In contrast, this ratio is inverted (ca. 1:40) for differentiated cells (E = −170 mV). This is in agreement with previously referenced independent data proving that active (reduced) HDAC8 is involved in cancer. Moreover, a gene expression analysis found genes involved in antioxidant defense overexpressed associated with clinical resistance in a vorinostat phase 1 trial [41] and drug resistance can be overcome by a combination with redox-modulating compounds in leukemia cell lines and primary leukemia cells [42]. These findings highlight a significant role for the redox environment in cancer cells and particularly drug resistance. Moreover, our data suggest a positive relationship between cancer and enzyme activity of HDAC8. However, differences in redox-potentials of whole cells do not display the real redox conditions in particular subcellular compartments and are unsufficient to predict the rate of interconversion between different redox partner molecules.

### 3.6 Oxidation kinetics of HDAC8

**Fig. 4:**
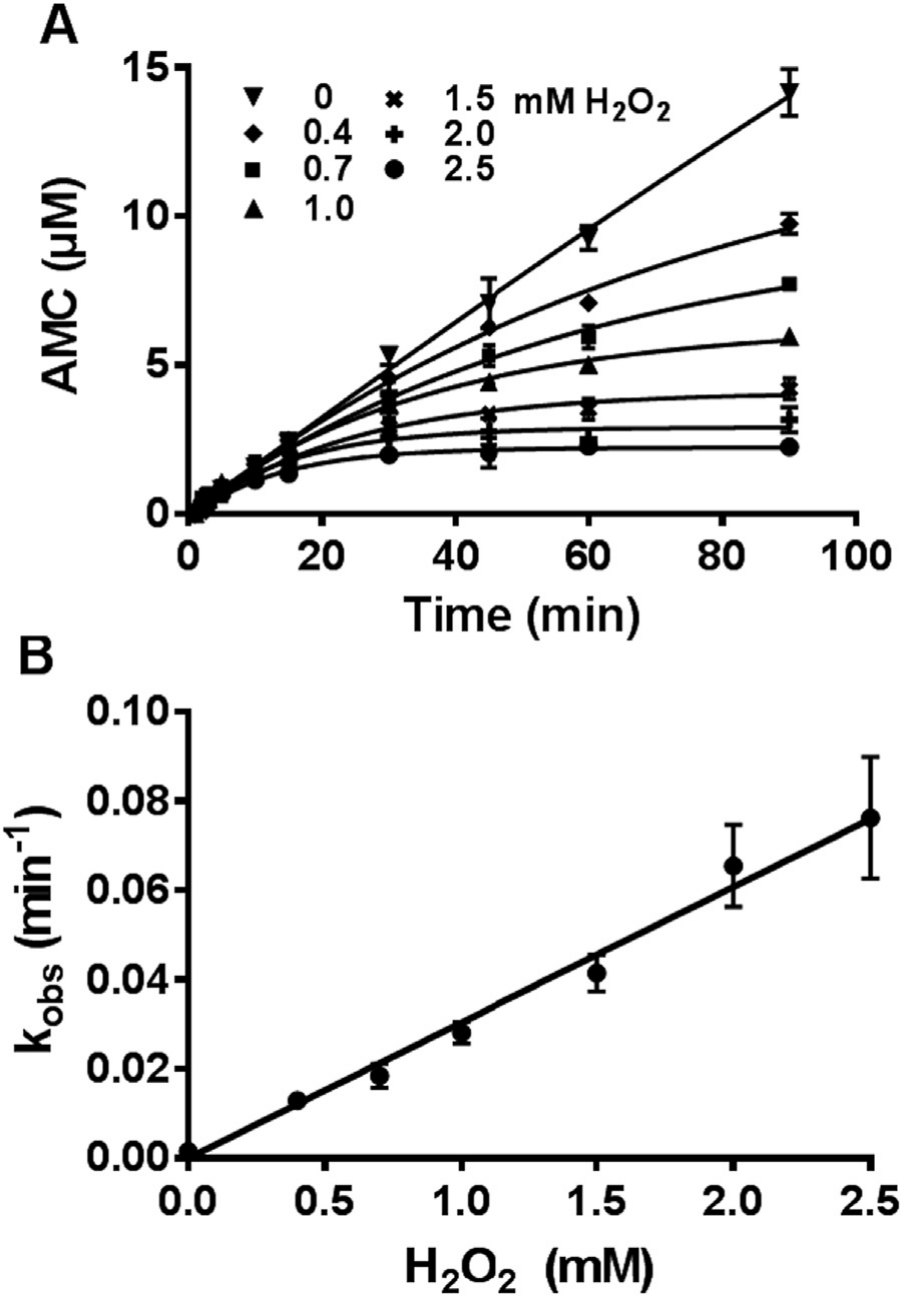
Oxidation kinetics of HDAC8_wt_: A) Enzyme kinetics of HDAC8_wt_ in the absence (filled down triangles) and in the presence of indicated concentrations of H_2_O_2_. Slow oxidation kinetics by H_2_O_2_ causes a time-dependent decrease of enzyme activity. Smooth curved lines represent mono-exponential fits. B) The observed pseudo-first order rate constants (k_obs_) obtained from A) are plotted versus different indicated H_2_O_2_ concentrations (ranging from 0 to 2.5 mM) used to oxidize HDAC8. The second order rate constant of the oxidation reaction is 0.51 ± 0.01 M^−1^ s^−1^. Data means and standard deviations are shown, n = 3.

Having shown that redox-switching of HDAC8 involves amino acids C102 and C153, is reversible and characterized by a more stable and enzymatically inactive oxidized form, we analyzed the redox properties, kinetics and mechanism of the redox-switch in more detail (Fig. 4). For these experiments it was of utmost importance to stop the oxidation process after the indicated time by neutralizing excess H_2_O_2_ by catalase. The observed oxidation rates, k_obs_, were obtained from exponential fits to the time-dependent enzyme activity of HDAC8 and analyzed as a function of H_2_O_2_ concentration. From the linear relationship, the specific rate constant of HDAC8 oxidation by H_2_O_2_ was calculated to be 0.51 ± 0.01 M^−1^ s^−1^ (Fig. 4). This rate constant is much slower than for the antioxidants peroxiredoxins, glutathione peroxidase 1 or catalase (10^5^–10^7^ M^−1^ s^−1^) and also slower than known redox-switches like PTP1B, SHP-2 or Keap1 (20–140 M^−1^ s^−1^) [43]. The small oxidation rate requires the molecular interaction with a redox transfer molecule such as a redox-protein to enable efficient redox regulation of HDAC8 under physiological conditions. On the other side, such a mechanism ensures selectivity through molecular recognition. H_2_O_2_ is a signaling messenger that can selectively modify cysteines in redox-regulated proteins, which react rather slowly with the free oxidant. Although thermodynamically favored, the oxidation of cysteines by H_2_O_2_ is kinetically hindered. In principle, the oxidation reaction can be accelerated catalytically, e.g. by thiol peroxidases [15], [44], [45]. It is currently widely accepted that peroxiredoxins are not only key protectors against oxidative stress [44], [46], [47]. There is growing evidence that peroxiredoxins are major sensors and transmitters of H_2_O_2_ signals [48]. It was shown that peroxiredoxins are able to transfer oxidizing equivalents to kinases, transcription factors and other redox-regulated proteins through specific protein-protein interactions [15], [45], [48], [49].

### 3.7 Physiological relevance of redox-regulation of HDAC8

To analyze the cellular impact of redox regulation of HDAC8, we used the BE(2)-C neuroblastoma tumor cell line known to depend on HDAC8 for cell growth and survival [22], [50], [51]. We investigated the putative redox-regulation of HDAC8 under physiological conditions by modulating the concentration of endogenous H_2_O_2_. NOX family members are reactive oxygen species (ROS)-generating enzymes that regulate redox-sensitive signaling pathways involved in cancer development and progression [52]. They produce high levels of superoxide and H_2_O_2_ in various cancer cell lines [52].

**Fig. 5:**
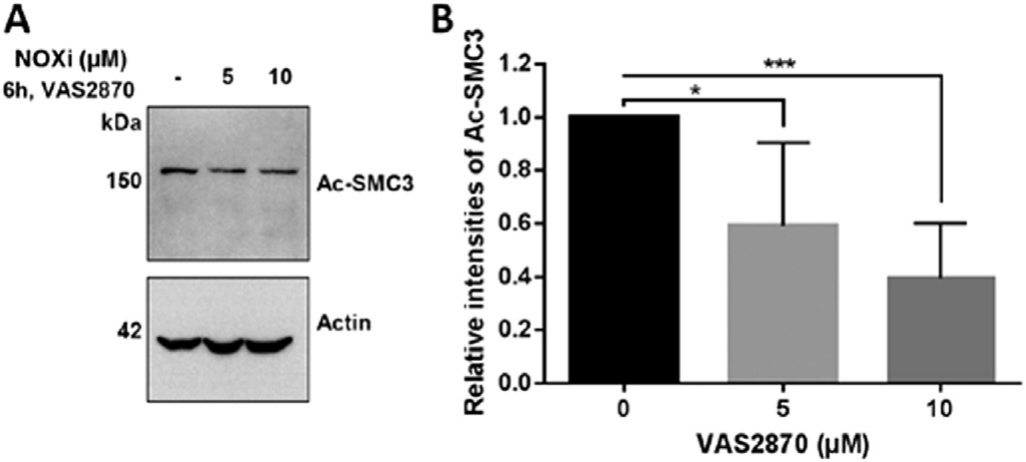
Inhibition of NOX in BE(2)-C cells by different indicated concentrations of NOX inhibitor VAS2870 leads to a lower intracellular H_2_O_2_-level and decreased acetylation of SMC3. Immuno-blot results (A) and their respective quantification (B) are shown. The solvent (DMSO) control is indicated with a hyphen sign. Data here are the average ± standard deviation of five independent repeats. Statistical significance was determined by Student's *t*-test. *: p-value = 0.019 and ***: p-value = 0.0002.

NOX activation and ROS production is also linked to the activation of oncogenes, such as RTKs (receptor tyrosine kinases) and their downstream signaling cascades (e.g. PI3K, mTOR, AKT) leading to tumor growth and survival [52]. In neuroblastoma, NOX enzymes are involved in retinoic acid-induced neuroblastoma cell differentiation [53]. Treatment of the BE(2)-C cells with NOX inhibitor VAS2870 reduces the intracellular production of H_2_O_2_ and concomitantly the acetylation of SMC3 [5] (Fig. 5). This demonstrates a relationship between the intracellular levels of ROS including H_2_O_2_ and HDAC8 activity. Together, these data suggest that HDAC8 could be redox-regulated under physiological conditions despite its very slow reaction kinetics with free H_2_O_2_. We hypothesize that the mechanism of redox regulation involves at least one H_2_O_2_ sensor and transmitter enzyme to accelerate specific oxidation of HDAC8. Interestingly, peroxiredoxin 6 has been described as a putative interaction partner and substrate of HDAC8 using a proteomic approach [54]. This raises the possibility that both proteins may form a redox relay, where oxidative equivalents are transmitted from peroxiredoxin 6 to HDAC8. However, the exact mechanism of intracellular ROS/H_2_O_2_ signaling to HDAC8 is unknown and currently under investigation.

## 4 Conclusions

We provide evidence that the enzyme activity of HDAC8 depends strongly on its redox state. The occurrence of a characteristic disulfide bond under oxidizing conditions is associated with a transition into a significantly more stable protein resulting in a complete but reversible loss of enzyme activity. C102 and C153 are clearly identified to be the integral components of the redox-switch in HDAC8. The physiologically relevant regulation of HDAC8 by redox signal transduction is suggested by a clear connection between the activities of ROS/H_2_O_2_ generating NOX and HDAC8 in neuroblastoma tumor cells. The redox-switch is only conserved among the members of HDAC class I suggesting that HDACs 1, 2 and 3 could also be regulated by a homologous switch. Further studies are required to obtain a full understanding of the redox-regulation network of human class I but also class II HDACs.
