# Supplementary material for: The enzyme activity of histone deacetylase 8 is modulated by a redox-switch
Source: Redox Biol. 2018 Sep 27;20:60–7. doi: 10.1016/j.redox.2018.09.013 (PMC6174833; doi:10.1016/j.redox.2018.09.013)
Supplement: Supplementary file 1 — Supplementary material [file mmc1.docx]

Supporting Information

The Enzyme Activity of Histone Deacetylase 8 is Modulated by a Redox-Switch

Niklas Jänsch^1^, Christian Meyners^1^, Marius Muth^1^, Aleksandra Kopranovic^1^, Olaf Witt^2,3,4^, Ina Oehme^2,3^, Franz-Josef Meyer-Almes^1,^*

^1^ Department of Chemical Engineering and Biotechnology, University of Applied Sciences Darmstadt, Haardtring 100, 64295 Darmstadt, Germany.

^2^ Preclinical Program, Hopp Children’s Cancer Center at NCT Heidelberg (KiTZ)

^3^Clinical Cooperation Unit Pediatric Oncology, German Cancer Research Center (DKFZ), INF 280, D-69120 Heidelberg, Germany and German Cancer Research Consortium (DKTK)

^4^Department of Pediatric Oncology, Hematology and Immunology, University Hospital Heidelberg, Heidelberg, Germany


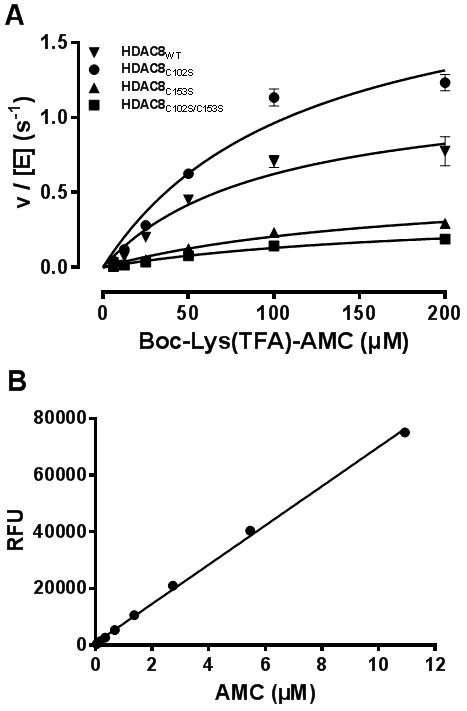


Fig. S1: Determination of the Michaelis-Menten-parameters of HDAC8 variants. (A) 10 nM of (HDAC8_WT_, HDAC8_C102S_) and 100 nM of (HDAC8_C153S_, HDAC8_C102S/C153S_) were incubated with increasing concentrations of Substrate at 21°C for 5 min. The enzyme velocity devided by the total enzyme concentration is plotted versus the concentration of the substrate. In each case, substrate turnover is less than 5%. The data points represent means and standard deviations, n=3. (B) Calibration curve for released fluorescence dye 7-amino-4-methyl coumarin (AMC).


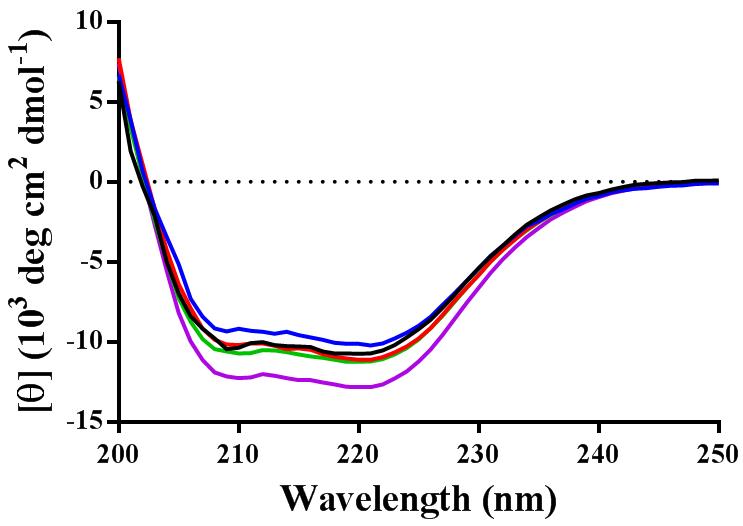


Fig. S2: CD-spectra of reduced (blue) and oxidized (black) HDAC8_wt_ enzyme. In addition, the spectra of the mutant proteins HDAC8_C102_ (red), HDAC8_C153_ (green) and HDAC8_C102/C153_ (magenta) are shown.


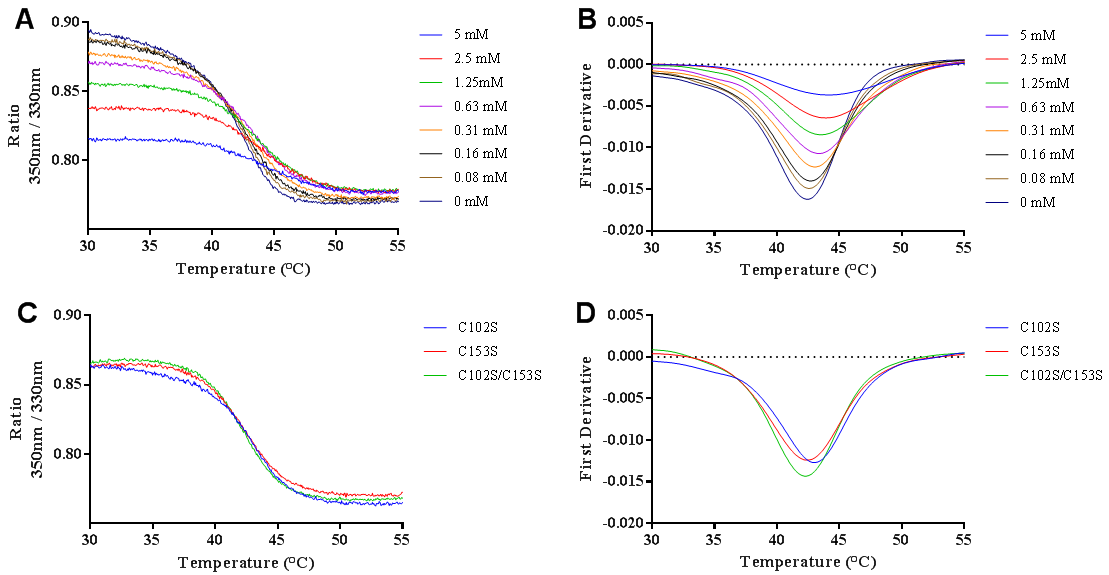


Figure S3: Differential scanning fluorometric curves: Ratio of tryptophan fluorescence at 350 and 330 nm versus temperature for HDAC8_wt_ in the presence of indicated concentrations of hydrogen peroxide (A) and various HDAC8 variants in the absence of hydrogen peroxide (C) are shown on the left side and the corresponding first derivatives of the melting curves, (B) and (D), are shown on the right side, respectively.


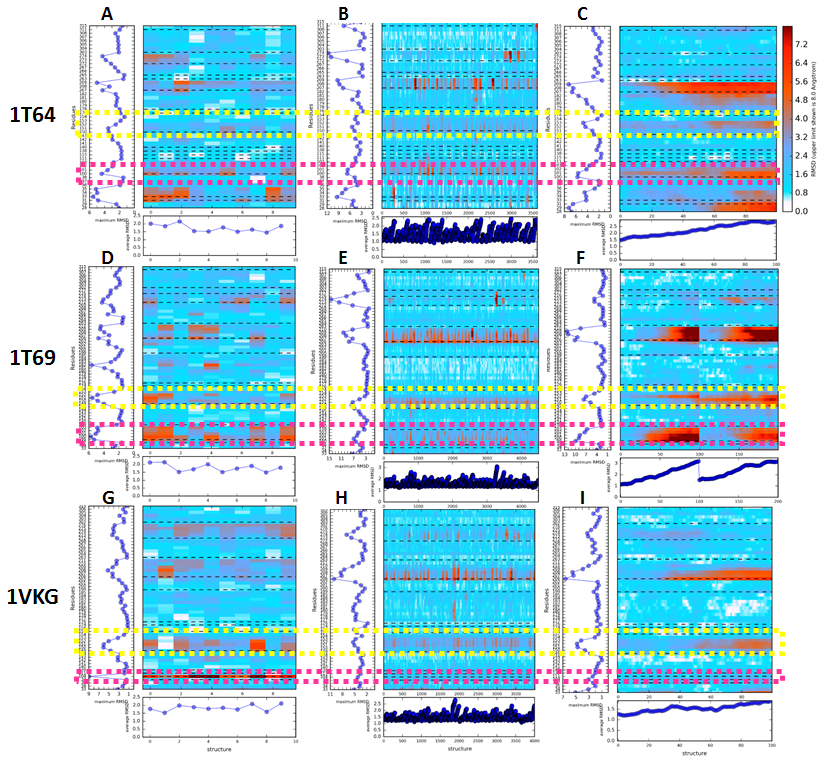


Fig. S4: Illustration of HDAC8 flexibility obtained from tCONCOORD (A, D, G), L-RIP (B, E, H) and RIPlig (C, F, I) simulations using the TRAnsient Pockets in Proteins (TRAPP) ^1^ webserver at the university of Heidelberg and applied to three different crystal structures of HDAC8/ligand complexes (PDB-ID’s: 1T64, 1T69, 1VKG). The dotted yellow boxes indicate the L3-loop and the dotted magenta boxes the L2-loop. The L2- and L3-loop show larger deviations (orange to red) of transient structures from the starting structure during simulations indicating regions of increased flexibility.

Detailed settings for above mention TRAPP simulations (Webserver: https://trapp.h-its.org/trapp):

For tCONCOORD 10 ensembles of protein structures were generated. For L-RIP all binding pocket residues were perturbed using 100 L-RIP pulses, 100 molecular dynamics steps in L-RIP pulses. For RIPlig the pseudo-ligand leucine was used for perturbation again using 100 L-RIP pulses, 100 molecular dynamics steps in L-RIP pulses, but limiting the generated trajectories to a maximum of 4 using 1 seed for pseudo-ligand position generation. Pocket analysis was performed in a radius of 7.5 Å around the ligands, respectively.


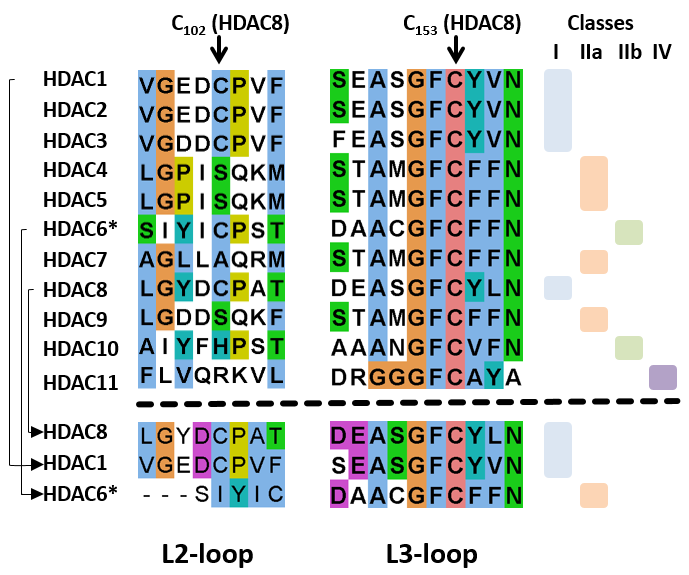


Figure S5: Multiple sequence alignments of L2- and L3-loop. Upper panel: multiple sequence alignment of all human class I, IIa, IIb and IV HDACs using the muscle algorithm in SeaView (http://doua.prabi.fr/software/seaview) ; lower panel: structure based sequence alignment using MOE 2016. * denotes the second HDAC domain of HDAC6. The amino acids in single letter code are colored using the clustal scheme in JalView.


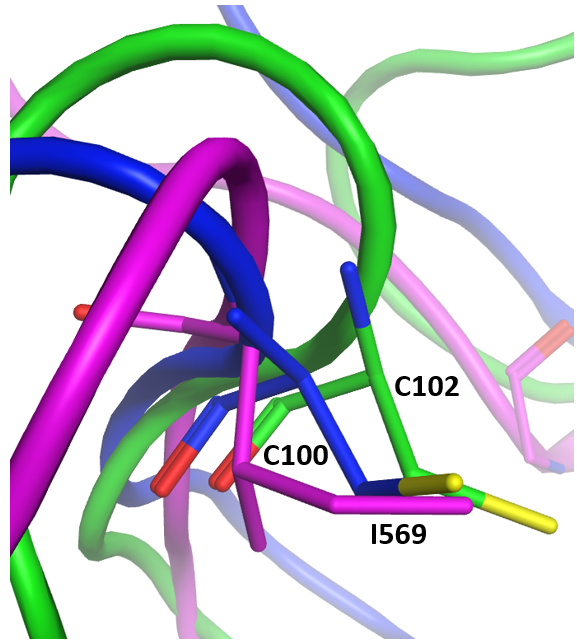


Fig. S6: Structural Alignment of HDAC1, HDAC6 and HDAC8. HDAC1 (PDB-ID: 4BKX) is colored in blue, the second domain of HDAC6 (PDB-ID: 5EDU) in magenta and HDAC8 (PDB-ID: 1T64) in green. The homologous amino acids to C102 (HDAC8) are C100 in HDAC1 and I569 in HDAC6.


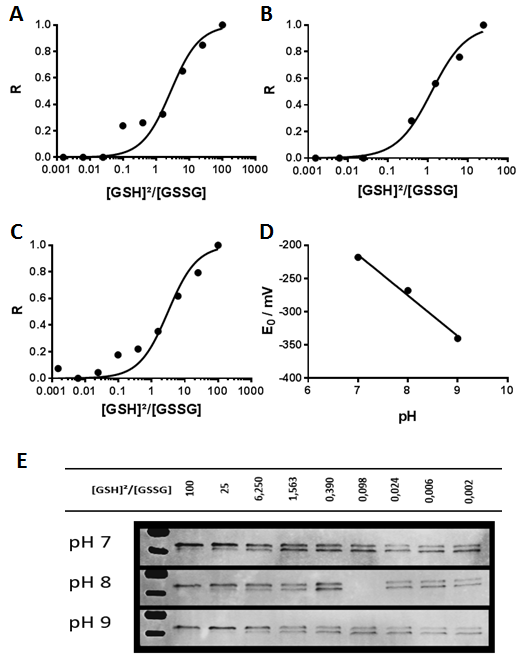


Fig. S7: Redox titrations of HDAC8_lowC_. A: pH7, B: pH8, C: pH9, D: pH dependency of redox potential, E: EMSA combined with western blot using anti-HDAC8-antibody as described under Materials and Methods (Determination of the redox-potential between Cys_102_ and Cys_153_) to quantify the very low amount of HDAC8_lowC_.

The equilibrium constant, K_eq_, of the redox-system (equation 1) was determined from a fit of equation 2 to the experimental data of a redox-titrations of HDAC8 (A/B/C). Then the standard redox-potential of the C102/C153-redox-switch was calculated using equation 3 for every pH-value.

HDAC8_red_ + GSSG ⇌ HDAC8_ox_ + 2 GSH (1)

$$\begin{matrix} \frac{[HDAC8_{red}]}{[HDAC8_{ox}]}=\frac{[GSH]^{2}/[GSSG]}{K_{eq}+[GSH]^{2}/[GSSG]} (2) \\ E_{HDAC8}^{0}=E_{GSH^{2}/GSSG}^{0}+\frac{RT}{nF}\ln K_{eq} (3) \end{matrix}$$

**References:**

1. Stank, A. et al. TRAPP webserver: predicting protein binding site flexibility and detecting transient binding pockets. *Nucleic Acids Res* (2017).
